# Malignant lymphoma of the oral cavity and the maxillofacial region: Overall survival prognostic factors

**DOI:** 10.4317/medoral.18903

**Published:** 2013-05-31

**Authors:** Janet O. Guevara-Canales, Rafael Morales-Vadillo, Sonia J. Sacsaquispe-Contreras, Carlos Barrionuevo-Cornejo, Jaime Montes-Gil, Carlos E. Cava-Vergiú, Fernando A. Soares, Henrique D M. Chaves-Netto, Maria G A M. Chaves

**Affiliations:** 1 Dentist, Master, Professor of the Faculty of Dentistry of the University de San Martín de Porres, USMP, Lima, Peru; 2 Dentist, Master, PhD, Professor of the Faculty of Dentistry of the Peruvian University Cayetano Heredia, Lima, Peru; 3 MD, Specialty in Pathology, National Institute for Neoplastic Diseases, Lima, Peru; 4 Dentist, Master, PhD, Professor of Dentistry of the University de San Martín de Porres, USMP, Lima, Peru; 5 MD, Master, PhD, Specialty in Pathology, Hospital A.C. Camargo, Sao Paulo, Brazil; 6 Dentist, Master, PhD, Professor of the Faculty of Dentistry of the Federal University of Juiz de Fora, Minas Gerais, Brazil

## Abstract

Objective: To identify the overall survival and prognostic factors of malignant lymphoma of the oral cavity and the maxillofacial region. Study Design: Clinical records data were obtained in order to determine overall survival at 2 and 5 years, the individual survival percentage of each possible prognostic factor with the actuarial technique, and the survival regarding the possible prognostic factors with the actuarial technique and the Log-rank and Cox’s regression tests. Results: Of 151 subjects, an overall survival was 60% at 2 years, and 45% at 5 years. The multivariate analysis demonstrated statistically significant differences for clinical stage (p=0.002), extranodal involvement (p=0.030), presence of human immunodeficiency virus (p=0.032), and presence of Epstein-Barr virus (p=0.010). Conclusion: The advanced clinical stage and the larger number of involved extranodular sites are related to a lower overall survival, as well as, the presence of previous infections such as the human immunodeficiency and the Epstein-Barr virus.

** Key words:**Lymphoma, oral cavity, survival.

## Introduction

Malignant tumors of the oral cavity are infrequent, representing only 5% of all those occurring in the human body. Among malignant tumors of the oral cavity, squamous cell carcinomas are the most frequent type (90 to 98%), and malignant lymphomas are the most outstanding among the remaining 2 to 10%. These lymphomas are neoplasms characterized by the clonal proliferation of lymphocytes and of their cell precursors (1), and of lymphocyte cell lines (2). The only feature shared by this group of is that they arise as the result of a somatic mutation of a lymphocyte progenitor (3).

ML are a heterogeneous group of neoplasms that are classically divided into two subgroups, Hodgkin Lymphoma (HL) and the non-Hodgkin Lymphoma (NHL) (2,4) due to their biological, histological, immunophenotypical differences, and clinical behavior patterns (1).

In general terms, NHL has a worse prognosis than HL, because when it is diagnosed, patients are often already at an advanced stage of the disease, and these neoplasms are more aggressive (5).

Over the years, many schemes of classification have been described the first based only in the cell morphology. In 1995 the World Health Organization (WHO) started the project of classification of haematopoietic and lymphoid-tissue tumors published in 2001. Since then, it has been accepted by most pathologists and clinicians as the first world system of consensus classification, which is based on the combination of morphological, immunophenotypical data, molecular genetics, and clinical aspects. The classification also helps predict the clinical aggressiveness of the subtype. The classification was re-edited by the WHO in 2008 with the participation from the Hematopathology Society and the European Association of Hematopathologists. A combination of morphological, immunophenotypical data, genetic characteristics, and clinical syndromes were included, in addition to defining new entities and giving solutions to diagnosis accuracy problems (6), this included the recognition of small clonal lymphoid populations and the identification of diseases characterized by the participation of certain anatomical sites or other clinical characteristics such as the age (7).

In general, HL corresponds to approximately 14% of all the lymphomas (8) and NHL to 86% of lymphomas (9,10). Usually, lymphomas first appear as nodal disease most commonly within the cervical and mediastinal nodes, extranodal lymphomas is not as common; whereas, NHL appears commonly in extranodal locations (10,11) between 24% (12) and 48% (11).

NHL occur in 3.5% of all the malignant neoplasms of the oral cavity and the jaw bones (8,13).

The causes of the NHL are uncertain (2). Risk factors include exposure to pesticides and radiation, long-term immunosuppresion, and autoimmune diseases such as the rheumatoid arthritis, systemic lupus erythematosus, and the Sjögren syndrome (14). Several viruses have been suggested as potential causes for this disease (2), including the Epstein-Barr virus (EBV), Human T-cell lymphotropic virus 1 (HTLV-1), Human immunodeficiency virus (HIV), Human Herpes virus type 8 (HHV-8, HVSK) (15) and Hepatitis B, C and G virus (HBV, HCV and HGV) have all been related to a greater risk of NHL (16) and can act in multiple lymphocyte cell clones, contributing to their neoplastic transformation (17). Other microorganisms involved in the genesis of the NHL are Helicobacter pylori and the Chlamydia (16).

In the oral cavity NHL corresponds to its extranodal presentation (8) and can occur in the soft tissues or bones. The most common sites being the soft tissues of the oral cavity, the palatal mucosa, the gingiva, the tongue, the cheek, the floor of the mouth and the lips and in the maxillofacial region, the salivary glands and the maxillary sinuses (8).

The systemic signs and symptoms include fever of an unknown origin (> 38ºC), inexplicable weight loss (> 10% of the body weight in the last 6 months before admission), night sweats (1,18), visceral pain and malaise (“B” symptoms), identified in 40% of the new cases (18). Patients with oral lymphoma rarely present withfever or weight loss (8,19) and therefore they are almost never accompanied by “B” symptoms (13,19).

The classification of the International working formulation for clinical usage (20) grouped NHL according to their increasing aggressiveness (low level, intermediate level and high level). Nevertheless, its usage has been gradually abandoned and replaced by the WHO’s classification.

Oral lesions of NHL can simulate inflammatory processes. The first important factor in the diagnosis of extranodal lymphoma in the oral cavity is to determine if the lesion is a tooth abscess, a periodontal infection a primary lymphoma of the oral cavity, or a more widespread manifestation of the disease (17).

In the region of the head and the neck NHL must be considered as a differential diagnosis when there is an inexplicable toothache, insensitivity, tooth mobility, increase of volume, ulceration, mass in an extraction alveolus or ill-defined lytic bone alteration (17).

The system most widely used for the clinical stage of ML is Ann Arbor’s based on the anatomical extent of the lesion, the number of tumor sites (nodal and extranodal), and the location (8). NHL staging is important for the therapy as well as to determine prognosis.

Several studies have tried to identify prognostic factors for the NHL. The International Prognostic Index (IPI) is the most widely used (21), and it incorporates several parameters that have been developed and validated in order to provide relevant prognostic information. These parameters are (22): age ≥ 60 years old, clinical stage (III or IV), the number of extranodal site involvement of > 1 site, performance status ≥ 2 and elevated serum concentration of Lactate Dehydrogenase (LDH).

Few NHL survival and prognostic factor studies have been carried out in the oral cavity. Since dental surgeons play an important role in the early detection of ML, they should be familiarized with the clinical and pathological characteristics of this disease in order to have a proper diagnosis. On the other hand, measuring and interpreting the characteristics of an ML already in place is essential to predict the survival of the patient. This study aims to identify the overall survival (OS) and prognostic factors of ML of the oral cavity and the maxillofacial region.

## Material and Methods

The design of the present study is of observational, descriptive, transversal, and retrospective in type. Subjects included in this study were ones who obtained a primary diagnosis of lymphoma of the oral cavity or the maxillofacial region seen in the Cancer Hospital, A. C. Camargo, São Paulo, Brazil, over the period of January 1980 to December 2005. The diagnosis of lymphoma was confirmed histologically and immunophenotypically, with almost 42% (63 cases) of the paraffin blocks being re-evaluated, while in other cases, their equivalent diagnoses were given according to Harris et al. (23). Clinical records with incomplete data were excluded.

In the analysis OS was determined and defined as the percentage of subjects who remain alive over the period comprised between the beginning of the treatment to the last visit to the doctor´s office, or date of death in years.

For the analysis of prognostic factors the following variables were considered: age, gender, presence of HIV and EBV, signs and symptoms, location, size, WHO’s diagnosis, histological type of malignancy, clinical stage, IPI, performance status, serum concentration of LDH, extranodal involvement, treatment, follow-up condition, and follow-up time.

Statistical analysis

The collected data were transferred to a Microsoft Excel program. Then the analysis was carried out with the assistance of the statistical program SPSS (Statistical Package for Social Sciences) version 17.0 for Windows (SPSS, Chicago, IL, USA).

OS analysis was calculated through two statistical tests: 1) the actuarial technique (mortality tables) in order to find the OS percentage at 2 and at 5 years and the individual survival percentage of each possible prognostic factor 2) Kaplan Meier’s li-mit-product method to determine the survival regarding each possible prognostic factor.

The analysis of prognostic factors was performed in two ways: 1) univariate analysis, with the Log-rank test (Mantel-Cox) to determine the individual statistical significance of the survival differences of Kaplan Meier’s limit-product and 2) multivariate analysis, with Cox’s regression model, being all the variables considered.

All values found through the different statistical tests were considered with significance from 0.05 (p < 0.05).

Tables and figures were elaborated in the same Microsoft Excel program. The analysis was carried out in a computer with Operative System Windows XP (Washington, USA).

## Results

Between January 1980 to December 2005, 3513 new cases of lymphomas were found, of which 4.3% (151) occurred at the level of oral cavity and maxillofacial region. Of these 151 lymphomas, the largest number (27.81%) occurred in patients 61 to 70 years old, and 53.64% were male. Tonsil was the most frequent location (43.05%), followed by parotid gland (13.91%) and palate (8.61%).

**Figure 1:**
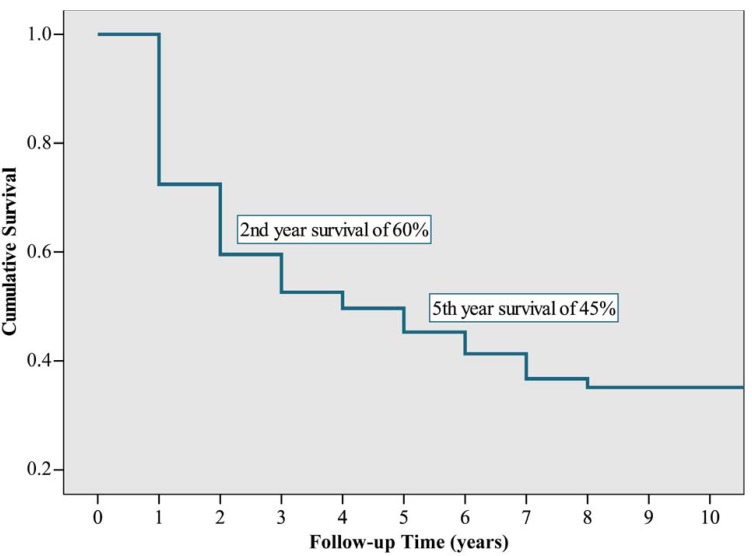
Overall survival curve for patients with lymphoma of the oral cavity and the maxillofacial region, seen in the Cancer Hospital, A. C. Camargo, over the period between January 1980 and December 2005.

An overall survival was found at 2 years of 60% and at 5 years of 45%, of the 151 lymphomas tested (Fig. 1).

In  Table 1 the OS at 2 years and at 5 years are described in detail with corresponding possible prognostic factors.

**Table 1:**
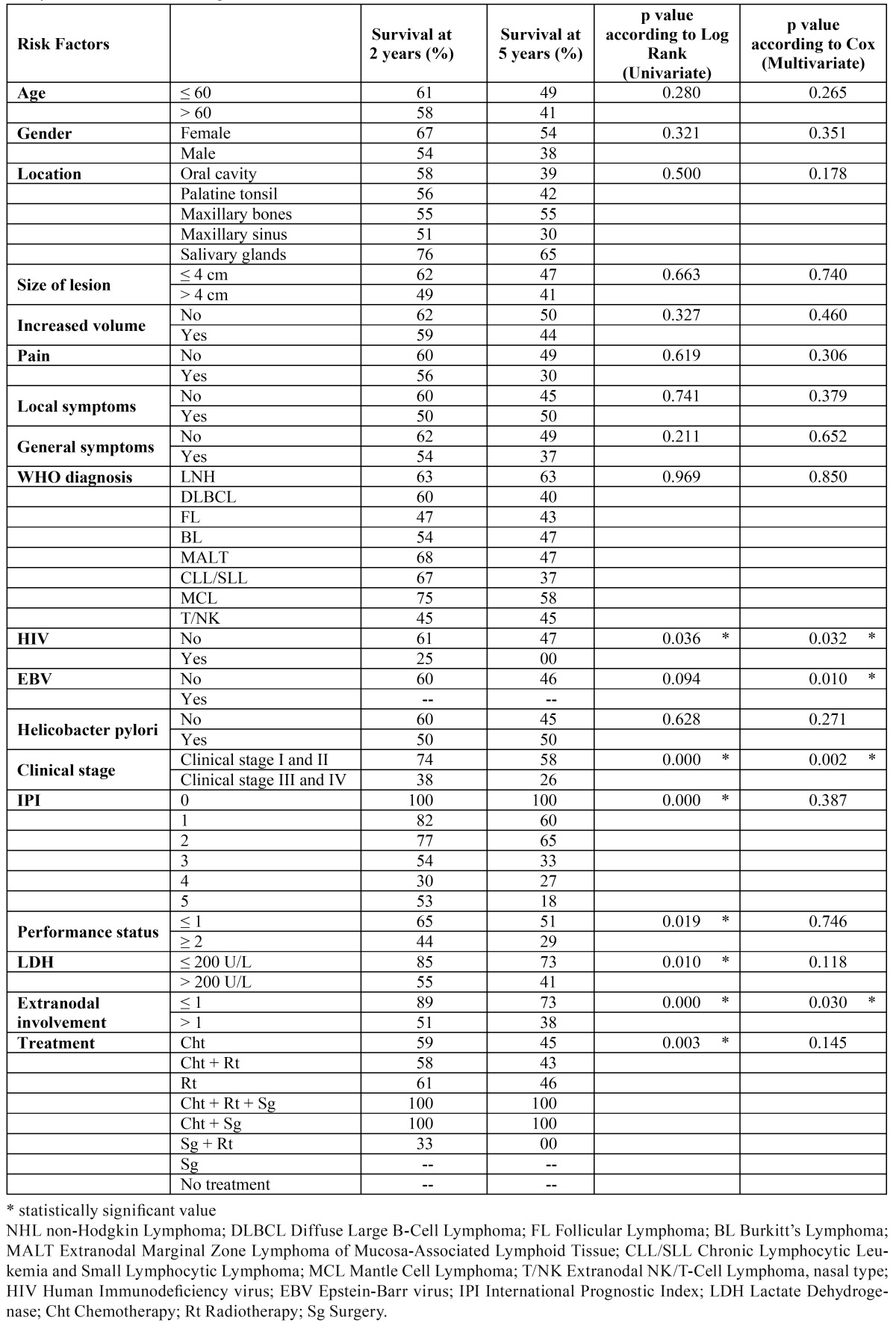
Survival percentage at 2 and 5 years evaluated for possible prognostic factors in subjects with lymphoma of the oral cavity and the maxillofacial region.

**Figure 2:**
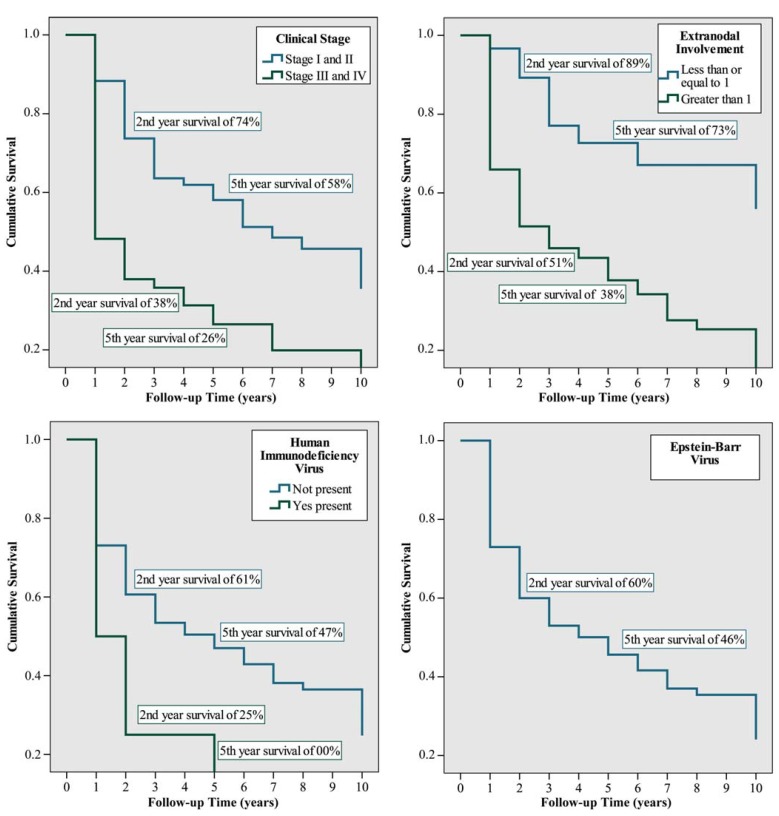
Mortality tables, for subjects with lymphoma of the oral cavity and the maxillofacial region, seen in the Cancer Hospital, A. C. Camargo, over the period between January 1980 and December 2005.

Survival of 74% and 58% was found at 2 years and at 5 years in subjects in stage I or II disease, and 38% and 26% in stages III and IV disease, respectively, which had a statistical significance in a univariate as well as a multivariate analysis. Subjects with only one affected extranodal site had survival rates of 89% and 73% at 2 and 5 years, respectively; whereas, subjects with a greater number of sites involved had survival rates of 51% and 38%, respectively. These values were also statistically significant in a univariate, as well as, multivariate analysis. The presence of HIV was also evaluated as a possible prognostic factor regarding survival, and values of 61% and 47% at 2 years and 5 years, respectively, were found in patients without HIV. Alternatively, patients with HIV infection had a survival at 2 years of 25%, but survival over 5 years was not observed. The differences found were statistically significant in a multivariate analysis. Presence of EBV was also evaluated with regard to survival; a survival at 2 years of 60% and at 5 years of 46%, respectively was found in non-infected subjects. Data from infected subjects were censored, therefore percentages of survival could not be obtained. With regard to this variable, there was no statistically significant univariate difference, but multivariate difference was found (Fig. 2).

The accrued proportion of survival up to 10 years is described in  Table 2. The risk factors influencing survival according to the adjusted model of Cox’s test (Hazard Ratio) showed that patients with a clinical stage I or II have 0.531 times more probability of survival than patients with a clinical stage III or IV. Patients with a number of extranodal sites involved less than or equal to 1 have 0.586 times more probability of survival than patients with a number of extranodal sites involved greater than 1. Patients with no presence of HIV infection have 0.678 times more probability of survival than patients with HIV infection; and the patients with no presence of EBV infection have 0.937 times more probability of survival than patients with EBV infection. These data are shown in  Table 3.

**Table 2:**
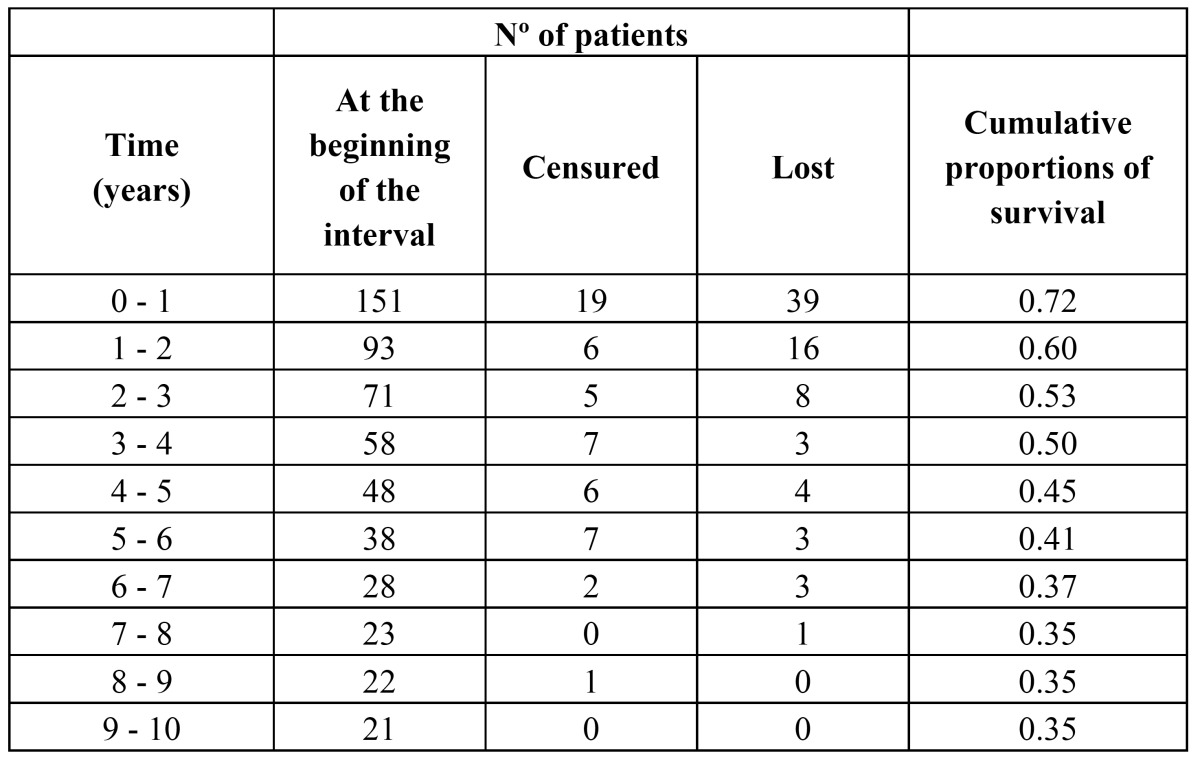
Cumulative survival for subjects with lymphoma of the oral cavity and the maxillofacial region.

**Table 3:**
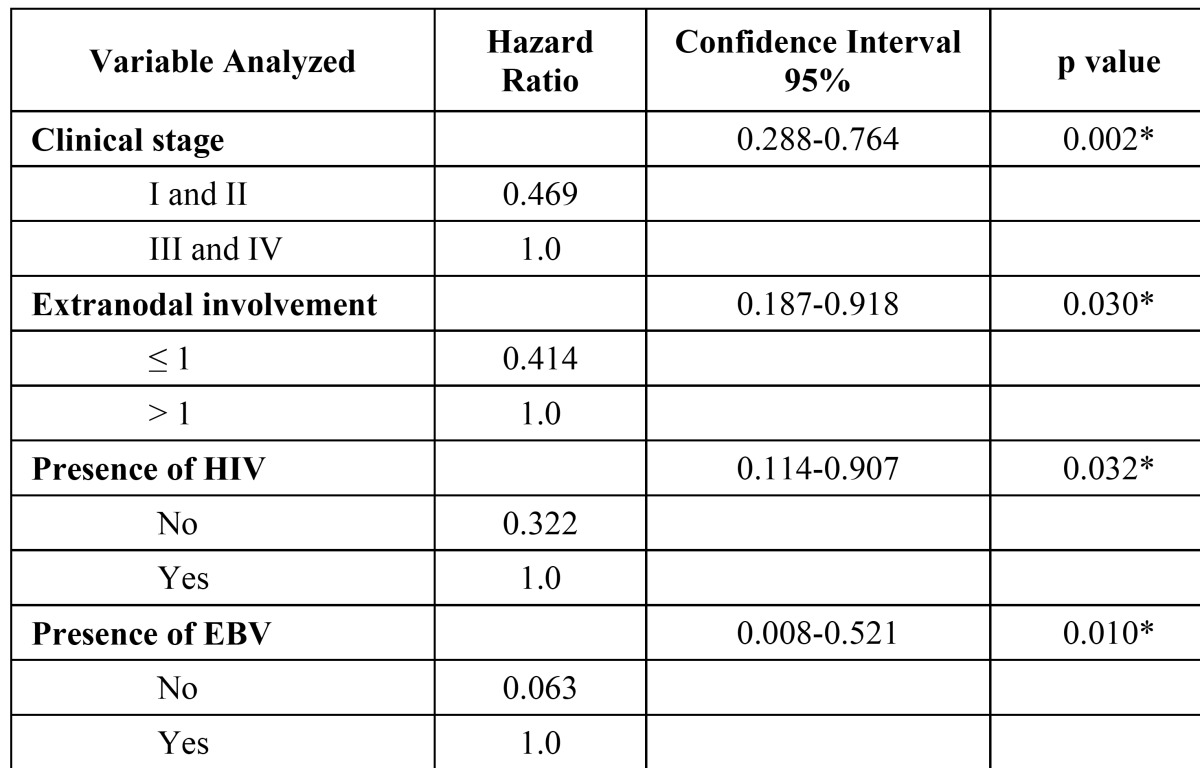
Analysis of potential risk factors associated with survival for subjects with lymphoma of the oral cavity and the maxillofacial region, according to multivariate Hazard Ratio of Cox analysis.

## Discussion

Regarding lymphomas found in the head and the neck 2.2% of them are primarily in the oral cavity or the maxillofacial region. Of intraoral malignant lesions 3.5% are lymphomas (8). All of our 151 study subjects were NHL. The percentage of OS found from the cases in this study was 60% and 45% at 2 and 5 years, respectively. When comparing the value obtained at 5 years, OS was much lower than the rate of 73% found by Rowley et al. (24) for HL and 65% for NHL extranodal found by Economopoulos et al. (25) Perhaps, this is due to fact that both studies were carried out in the head and the neck with a greater probability of cervical node level involvement.

In this series, no statistical significance was found in an univariate, as well as, multivariate analysis of OS comparing age, gender, signs and symptoms, size, WHO’s diagnosis, and/or histological type of malignancy.

In the present report there was a better OS in lymphomas located in the salivary glands, this could be related to the fact that in these cases the neoplasm tends to be more localized (65% at 5 years, in comparison with the locations in maxillary bones, palatine tonsil, soft tissues of oral cavity and maxillary sinus, which presented OS at 5 years of 55%, 42%, 39% and 30%, respectively). Nevertheless, these differences were not statistically significant.

Regarding the specific diagnosis as in other studies (26) no significant differences were found with regard to OS, although it has been reported that diffuse large B-cell lymphoma (DLBCL) had a survival at 5 years of 30%, whereas in the follicular lymphoma (FL) OS was reported as 70% (27). This could be related to actual therapeutic schemes that have improved the OS of aggressive NHL.

In Angiero et al. (17) and Economopoulos et al. (25) studies it has been reported that prognosis is influenced by the histological level of aggressiveness. In the last study mentioned, patients with low-level NHL had a 5 year survival rate of 83% compared to 52% for subjects who had the high-level histological subtype. In the present report no statistical significance was found at the histological level. In recent years, the WHO classification of hematological neoplastic disease has been reported individually and each has differing clinical factors and evolution. So these diseases are not grouped according to their aggressiveness.

Several reports have found that the survival prognosis gets worse with clinical stage (3,17,28). This is in line with the present study in which patients present a lower survival percentage as the clinical stage increases (74% and 58% at 2 and 5 years in stages I or II, respectively, and of 38% and 26% in stages III or IV), both individually and jointly, with statistically significant differences.

Mok et al. (22) found 5-year survival rates for nodal lymphoma and extranodal lymphoma of 57.4% and 52.1%, respectively. Moreover, the number of extranodal sites was not a significant predictive variable (p = 0.805). In the present study; however, the number of extranodal sites involved had a significant relevance with a p < 0.000 value when this criteria was evaluated in a univariate analysis and a p = 0.030 value when evaluated with multivariate analysis. A survival of 73% at 5 years was found when the subjects presented with an extranodal site affected and at 38% when the patient presented with more than one extranodal site involved. The difference can be explained by the statistical analysis because the variable is influenced by clinical stage.

In this study, the survival at 5 years in patients with normal serum concentration of LDH was 73% and 41% when LDH was elevated, these values were statistically significant when evaluated with a univariate analysis. These results are similar to those obtained in the Mok et al. (22) study in which the rates of survival at 5 years for the subjects with extranodal lymphoma who had normal and abnormal LDH were 77.3% and 44.9%, respectively (p < 0.001). These data confirm the value of LDH as a prognostic factor.

Treatment had an influence on OS (p = 0.003) in a univariate analysis. Based on a review of 53 patients with NHL of the head and neck, Ruijs et al. (29), suggested that radiotherapy alone is the adequate treatment for localized lymphoma while chemotherapy is preferable for patients with disseminated lymphoma. According to Mok et al. (22) the chemotherapy treatment regime does not affect the OS of subjects with a p = 0.715 and a p = 0.41 respectively.

In the Tanaka et al. (30) study 104 subjects with lymphoma and HIV infection had an OS at 4 years of 35.8%, the variables with significant influence on OS: previous HIV infection and an intermediate-high risk value of the IPI. In this study, similar results were found; patients with HIV infection had a quite low OS of 25% at 2 years.

In brief, the following prognostic factors of survival in patients with lymphoma in the oral cavity and the maxillofacial region were found in this study: clinical stage, HIV infection, EBV infection, and the number of extranodal sites affected.

The knowledge of prognostic factors in ML of the oral cavity and the area of the head and neck is essential for tailoring therapeutic interventions for individual patients with NHL in the oral cavity. Future studies comparing survival comparing other factors such as molecular studies, immunophenotypic profiles, and viral responses in NHL in these regions may help the development of new therapeutic strategies.
